# Good is not meaningful?

**DOI:** 10.12669/pjms.38.7.6564

**Published:** 2022

**Authors:** Shabih Zaidi, Hasan Zaidi

**Affiliations:** 1 Shabih Zaidi, NHS-U.K., & SIVU; 2 Hasan Zaidi, Imperial College, London

## INTRODUCTION

Ethics is a branch of Moral Philosophy. It is as old as mankind itself. Even the caveman observed a code of conduct for owning or sharing a piece of land, cultivating it, hunting for food, building a shelter and so forth.

Medical ethics is embedded within medicine. And yet it has not been given its due status until recently. COVID-19 may have played a significant role in its recognition as a measuring tool of professionalism. We all know that ethics teaches us how to differentiate good from evil, but more importantly, ethics uncovers the practical illustrations of goodness during the pandemic.

One major lesson we learnt was the moral failure of governments and health authorities in telling the truth. Was telling the truth not good? Might telling the truth have embarrassed the authorities?

So here is a statement, ***‘good is not meaningful’***: Let us look at it from the point of view of Metaethics. While normative ethics defines the principles and parameters of what is good and what is evil, metaethics looks at the second-order non-moral questions, the semantics and epistemology of moral thinking and its relevant discussions.^1^ Central to metaethics is the debate between realism and anti-realism.

### This can be applied to the term ‘good’ as follows

Whilst anti-realists argue that ‘good’ refers to something independent of humans, realists argue for ‘good’ as related to how we respond to the world. Realists could argue that good is meaningful, disagreeing with the statement, as good can be understood as part of the natural world. Consequently, there is a debate as to whether the term ‘good’ can be known to us, with naturalists taking a moral realist position where ‘good’ equals a physical predicate – a position rejected by intuitionists who view moral truths as relativist, and emotivists who attach goodness to statements about our beliefs.

A vast array of emotions were witnessed during the pandemic. Therefore, we have chosen to argue for the Emotivist view, that good is meaningful, to be understood using our feelings.

Ethical naturalists hold that morals can be observed, objectively, using evidence. For example, a utilitarian may argue that something may be proven good if it brings the maximum good for the greatest number of people, as measured using the Hedonic Calculus – which argues nature has placed mankind under two sovereign masters, pain and pleasure.^2^

This theory is cognitivist, as by equating good to a natural property ‘good’ becomes meaningful as it can be known to all. This can be praised for all people have some sense perception, ensuring that everyone can know what is right *a posteriori* (proceeding from observation) – unfortunately, this is a view that is easily criticized. For instance, Hume’s Law^3^ states that you cannot go from an ‘is’ to an ‘ought’, a statement of fact to a moral. It is wrong to assume that things in the natural world are indicative of what is right, there is a jump in logic. This is further reinforced by ideas that sense-perception is flawed – one cannot often see a moral decision from multiple perspectives because one does not have the inductive experience to do so, making recognizing ‘good’ in first-time decisions difficult if not outright impossible.

The illogical nature of naturalism is also clear when one follows G. E. Moore’s Open Question Argument. Moore argues that it is a mistake to define moral terms with reference to other properties and break Hume’s Law.^4^ His objection explains how the view that moral properties exist in the world as natural properties can be disproven logically (for example, the properties of pleasure and goodness being equal).

If two objects are analysed in terms of each other and are equal, if one were to ask ‘does X = Y’, then the answer would be meaningless, a tautology, thus making it a closed question that reveals no new information. e.g. Are all bachelors unmarried men?’ If two concepts are analysed in terms of each other and are not equal, if one were to ask ‘does X =Y’ the answer would express a meaningful answer e.g. ‘no because…(followed by reasoning)’ providing new information – such are open questions that do not show a conceptual misunderstanding as the predicates are not the same.

Moore argues that if we ask if a natural property is good, it would be answered meaningfully, always leading to an open question - showing that the predicates of goodness and the natural world are not tautologous, thus cannot be equal – therefore, moral properties cannot logically equal natural properties. Consequently, it becomes clear that following an ethical naturalist view does not make ‘good’ meaningful as such theories are logically flawed and thus unconvincing. Instead, we could look to another theory which avoids the natural world to determine whether ‘good’ has meaning.

Perhaps realist Intuitionists come closer to finding the meaning of good – intuitionists hold the view that moral truths are not fixed and are not absolute. G. E. Moore within *Principia Ethica* (George Edward Moore, 1903),^4^ argues “Good is a non-definable property”, using an analogy; he writes: “We know what ‘yellow’ is, and can recognize it… in the same way we can know what ‘good’ means but cannot define it”. To better understand this, we can say goodness is like beauty, a quality found in things that cannot be defined or quantified. He justifies this view by arguing that good is self-evident (so meaningful) and a simple idea that cannot be broken down into simpler ideas. It is a sum of many parts which cannot be fragmented. He gives the example of ‘not’ as an easier-to-understand simple word, that cannot be defined further than the simple building block as we use it for defining other ideas.

### Ross on duties:

This was expanded on by W. D. Ross, who described six Prima Facie Duties, known intuitively, (a) Fidelity, (b) Gratitude, (c) Justice, (d) Beneficence, (e) Non-Maleficence, (f) Self-improvement. (in no particular order). Ross points out that the mature person intuitively knows what is good objectively, but that morals are conditional – whether they should be followed depends on one’s overriding duty - when a conflict between duties arises, one should follow the overriding duty.^5^ (Let us momentarily recall Maslow’s hierarchy of needs which are (a) physiological needs, (b) safety needs, (c) love and belonging needs, (d) esteem needs, and (e) self-actualization needs).

Ross’s view can be questioned, for one must assume what they can recognize intuitively is actually good. Nietzsche 6 argued what is good may be evil and what is evil may be thought to be good, for we could be ethically colour-blind. He points out that virtues could be vices as Christian virtues of humility and obedience are detrimental to the human spirit, individuality, and the evolutionary need for self-assertion, whilst vices may be virtues, for pride (condemned by Paul) is actually a virtue for humanity to remain dominant. Nietzsche and Machiavelli are two highly influential but controversial philosophers as a student of philosophy would know. Did many politicians and power brokers employ this philosophy in their decision-making from January 2020 onwards, believing that morality could be overridden by an overriding duty? Whilst Ross may be right in that we can intuitively know these six duties, it is unclear to Nietzsche that there is an objective assessment for right and wrong – so ‘good’ may not actually mean these virtues. Instead, Intuitionism offers the individual too much freedom and may suffer from anti-nomian problems where one is unsure what is actually ‘good’, and just assumes certain ‘virtues’ are – leaving open the opportunity for bad attributes to be recognized as good. Thus, like with ethical naturalism, intuitionists assume humans can recognize an external good, for which there is no evidence.

### Virtues and Vices:

Virtue shall always remain virtue and vice always vice. The best way to imbibe virtues in our lives as health professionals is to intuitively act good, avoiding evil, instinctively, repeatedly, habitually and imperceptibly. That is what virtue-based ethics advises. Virtue ethics was introduced by Plato, who defined its four pillars as Courage, Compassion, Practical wisdom (Phronesis) and Justice. Aristotle expanded the subject and reached a conclusion that virtues are good and they bring happiness. To Plato, the ultimate goal in life should be happiness. In turn, it leads to a state of mind called Eudaimonia (heart’s contentment, *itemenan e qalb)*.

But there is another argument. Emotivists hold the view that moral statements are not statements of fact but instead either express beliefs or emotions, with no cognitive, knowledge content. Thus, they do not have a conventional meaning in being true or false. Instead, they describe sentiment - like Intuitionists. The emotivists agree that morals are absolute and observable but also argue that morality is not a fact but entirely relative, for logical positivists (like Ayer) reject the existence of things that cannot be known through verifiable science.

### Moral Relativism:

Moral relativism is a subject in its own right.^7^ It is a highly controversial but important subject in Metaethics as well as moral philosophy. Fundamentally it means that moral values are not absolute but relative to circumstances, faith, culture, values and practices. Different societies may have varied beliefs and practices such as polygamy, female genital mutilation, or same-sex marriages. What is acceptable in one society may be abhorrent for another.

### Emotivism:

Emotivism is influenced by Hume’s (1711-1776) views that morality cannot be proven empirically, rather it is a question of personal sentiments. He wrote about the action of murder, which many view as bad (a vice). He writes, “the vice entirely escapes you, as long as you consider the object. You will never find it, till you turn your reflection into your own breast, and find a sentiment of disapprobation, which arises in you, towards this action. It lies in yourself, not the object”. In other words, morality is in fact a display of one’s emotions or sentiments. Hume categorized two kinds of knowledge, analytic statements (tautologies) that explain the ‘relation of ideas’ (formal, abstract knowledge e.g. mathematics and logic), and synthetic statements (empirical statements) that explain ‘matters of fact’, derived from sense perception, empirical knowledge.

Ayer’s Verification Theory limits meaningfulness to these two types of statements – since ethical statements are neither. They lack meaning and so there are no fixed moral truths. Whilst one can criticize the Verification Principle for itself being unverifiable (as it determines statements are meaningful if either tautological or empirical, and it is neither – so is itself meaningless), this criticism ignores Ayer’s Logical Positivism – which holds this to be true. Instead, one could criticise emotivism for confusing ‘meaning’ and ‘use’. Abdollahi and Shirvand 8 have critically analysed this theory in an excellent paper.

### Virtue Ethics:

Alisdair McIntyre (b1929) revived virtue ethics after a thousand years or more of its eclipse, arguing that the meaning of moral statements varies on the occasion and cannot be applied universally, and that the same action can evoke different emotions in different scenarios. Instead, it is useless to hold beliefs as ‘good’ as there is no consistency. Nevertheless, this is not a vice but a virtue of the theory.

We witnessed a thousand displays of emotions by health workers, patients, teachers, pupils and the public at large. Phillipa Foot (1920-2010), a renowned philosopher, attempted to criticise emotivism by arguing that since emotions are based on beliefs then if shown inaccurately one can change another’s emotions.

This can be praised for allowing morals to change in order to remove beliefs that evoke negative emotions, avoiding persecution of others. Whilst good has no inherent meaning, by not appealing to another inaccessible world this theory is supported by Occam’s Razor, (Ockham’s) razor is a principle attributed to the 14^th^-century logician and Franciscan friar William of Ockham) which contends the simplest theory is most likely to be true. Therefore, good holds meaning as emotions, as this is simplest.

Mankind faced a thousand dilemmas during the pandemic. Plenty of good emerged from evil. No one knew how to react to this crisis. Health workers perished while performing a goodly act. Parents and teachers sustained moral trauma^9^ as they could not do good for their children, teachers failed as they could not do good for their pupils, and some states felt the same.

### Deontology and Consequentialism:

The lessons in terms of application of Deontology were many, as duty consciousness was seen abundantly. But what we saw more was the application of Utilitarianism such as in Italy in March 2020. But the finest example of doing good was the hands-on application of virtue ethics right across the globe.

Bentham and Mills’ utilitarian Consequentialism was seen in practical form during the early days of the COVID pandemic. Oxygen was in short supply, so the authorities expanded the supply to the largest number of people, rationing it on the basis of who needed it most rather than confining it to only a few. We also saw the shortage of beds more prevalent in developing countries, where patients in desperate situations shared not just their beds but also make-shift arrangements like cots or stretchers.

We observed the courage of the frontline health workers, who jumped into the wildfire without personal protective equipment in the first few weeks of the pandemic. We saw their wise decisions in the allocation of ventilators based upon needs rather than age or affordability. We also saw the display of justice in deciding upon sharing the vaccines, workload, or resources, based upon the ancient doctrine of Distributive justice.^10^

As mentioned before, Morality is universal^11^ It may have a shadow of colour and culture relative to different situations, but truth, honesty and loyalty will always remain the same. They may however be translated differently by societies justifying relativism.^12^

The pandemic has shown the importance of doing good, in health care as well as in our daily lives. Humanising medicine^13^ through the inclusion of humanities, philosophy, and particularly ethics in medical curricula to produce virtuous physicians who know the meaning of good and apply it in day-to-day practice^14^ is what the town crier is calling for.

## CONCLUSION

In conclusion, we disagree with the statement – ethical naturalism fails to convince us that good holds meaning in the world as it is illogical, and intuitionist theories make a jump in assuming humans can understand what is good in another external reality. Emotivism is not absolutist, so whilst ‘good’ may be viewed as lacking meaning as beliefs are neither synthetic nor analytic statements, ‘good’ holds meaning in our beliefs. To do good is part and parcel of human nature and integral to medical professionalism.
